# Efficacy and Safety of Epidermal Growth Factor Receptor (EGFR) Inhibitors Plus Antiangiogenic Agents as First-Line Treatments for Patients With Advanced *EGFR*-Mutated Non-small Cell Lung Cancer: A Meta-Analysis

**DOI:** 10.3389/fonc.2020.00904

**Published:** 2020-06-25

**Authors:** Fei Chen, Naifei Chen, Yu Yu, Jiuwei Cui

**Affiliations:** Cancer Center, The First Hospital of Jilin University, Changchun, China

**Keywords:** epidermal growth factor receptor inhibitors, angiogenesis inhibitors, non-small cell lung cancer, *EGFR* mutation, anti-VEGF, targeted treatment, first line, meta-analysis

## Abstract

**Background:** Tyrosine kinase inhibitors (TKIs) are standard treatment options for non-small cell lung cancer (NSCLC) with epidermal growth factor receptor (*EGFR*) mutations. Increasing clinical investigations have explored the value of EGFR-TKIs plus antiangiogenic drugs as the first-line treatment for *EGFR*-mutated NSCLC.

**Methods:** We systematically searched PubMed, Cochrane Library, and EMBASE for randomized controlled trials (RCTs) investigating EGFR-TKIs administered with or without antiangiogenic agents for advanced *EGFR*-mutated NSCLC. The latest RCT that was presented orally at the 2019 European Society for Medical Oncology Congress was obtained online. The endpoints included progression-free survival (PFS), overall survival (OS), objective response rate (ORR), disease control rates (DCRs), and grade 3 or higher adverse events (AEs).

**Results:** We included seven articles on five trials with 1,226 patients. The interventions for the experimental group were the first-generation EGFR-TKI erlotinib combined with bevacizumab (four studies) or ramucirumab (one study), and erlotinib monotherapy (four studies) or erlotinib plus placebo (one study) for the control group. All studies reached their primary study endpoints (i.e., PFS). Compared to erlotinib monotherapy, erlotinib plus antiangiogenic agents remarkably prolonged PFS [hazard ratio (HR) = 0.59, 95% confidence interval (CI) = 0.51–0.69, *P* = 0.000]; however, ORR, DCR, and OS were similar between the two groups. The overall grade 3–5 AEs increased in combination group (OR = 5.772, 95% CI = 2.38–13.94, *P* = 0.000), particularly the incidence of diarrhea (OR = 2.51, 95% CI = 1.21–5.23, *P* = 0.014), acneiform (OR = 1.815, 95% CI = 1.084–3.037, *P* = 0.023), hypertension (OR = 6.77, 95% CI = 3.62–12.66, *P* = 0.000), and proteinuria (OR = 13.48, 95% CI = 4.11–44.22, *P* = 0.000). Additionally, subgroup analysis demonstrated that Asian patients could significantly benefit from combination therapy (HR = 0.59, 95% CI = 0.50–0.69, *P* = 0.000). Patients with *exon 19* deletions (HR = 0.61, 95% CI = 0.49–0.75, *P* = 0.000) and *21 Leu858Arg* mutations (HR = 0.59, 95% CI = 0.47–0.73, *P* = 0.000) had almost equivalent PFS benefits when treated with double-blocking therapy. Patients with brain metastases at baseline in the combination group had a trend toward better PFS (HR = 0.55, 95% CI = 0.30–1.01, *P* = 0.001).

**Conclusions:** Erlotinib plus bevacizumab or ramucirumab in EFGR-mutated NSCLC first-line setting yielded remarkable PFS benefits; however, this was accompanied by higher AEs. Epidermal growth factor receptor–TKI plus antiangiogenic agent therapy may be considered a new option for advanced *EGFR*-mutated NSCLC patients.

## Introduction

Lung cancer is the most common malignant tumor. In addition, it has the highest morbidity and mortality worldwide (1). Approximately 85% of primary lung cancers are non-small cell lung cancer (NSCLC). Epidermal growth factor receptor (*EGFR*) mutations are found in 11–16% of lung adenocarcinomas in western countries and 50% in Asia (2). Since 2014, EGFR tyrosine kinase inhibitors (EGFR-TKIs) have become the standard first-line therapy for NSCLC patients harboring *EGFR*-activating mutations (3, 4). When treated with first- or second-generation TKIs, the median progression-free survival (PFS) is ~1 year, whereas with the third-generation TKI osimertinib, it is 18.9 months (5–7). Despite the high disease control rates, almost all patients eventually experience acquired resistance-mediated disease progression. Therefore, new combination regimens are needed to delay or overcome acquired TKI resistance. Neovascularization plays an essential role in supplying tumors with oxygen and nutrients, eliminating metabolic waste and facilitating tumor growth, progression, and metastasis (8–10). Hypoxia-driven vascular endothelial growth factor (VEGF) is a primary angiogenesis regulator that can activate proangiogenic signaling by binding to the vascular endothelial growth factor receptor (VEGFR) (11). Preclinical studies have shown that the EGFR signaling pathway can up-regulate VEGF expression (12–14). Epidermal growth factor receptor and VEGF share a common downstream signaling pathway. Further, VEGF/VEGFR plays a role in EGFR-TKI resistance (15–21). Antiangiogenic agents can increase the delivery of antitumor drugs by normalizing the blood flow of tumor blood vessels (22, 23). Lower doses of EGFR-TKIs were associated with earlier resistance (24, 25). Blocking the VEGF/VEGFR signaling pathway can abrogate EGFR-TKI primary or acquired resistance (15, 26). Increasing trials have sought to determine whether EGFR-TKIs plus antivascular drugs could elicit stronger antitumor effects than EGFR-TKIs alone for *EGFR*-mutant advanced NSCLC patients in the first-line setting. Therefore, we aimed to synthesize published randomized controlled trial (RCT) data to assess the efficacy and safety of this emerging remedy and to provide guidance for clinical decision-making and future researches.

## Methods

This meta-analysis was conducted in conformity with the PRISMA (Preferred Reporting Items for Systematic Review and Meta-Analysis) guidelines (Table S1).

### Search Strategy

PubMed, EMBASE, and Cochrane Library databases were searched to identify potential studies up to November 2019 that assessed the use of EFGR-TKIs plus angiogenesis inhibitors as a first-line therapy for NSCLC. Mesh and entry terms were both used for a comprehensive lookup. Additionally, the reference lists of included studies, and several commonly prescribed drugs were checked to further derive qualified publications. Further, we searched online for recently updated reports presented at meetings only and not presented in the databases (Supplementary Material).

### Inclusion Criteria

We included studies that met the following conditions: (1) recruited patients with cytologically or histologically confirmed advanced *EGFR*-mutant NSCLC; (2) compared EGFR-TKIs plus antiangiogenic drugs with EGFR-TKI monotherapy in the first-line setting; (3) reported outcomes: PFS, overall survival (OS), objective response rate (ORR), disease control rate (DCR), and grade ≥3 adverse events (AEs); and (4) were designed as RCTs. Articles or abstracts lacking any of the above criteria were excluded.

### Data Extraction

Two investigators (Fei Chen and Naifei Chen) extracted the main characteristic information, including the study name, sample size, median age, sex percentage, disease stage, treatment regimens, and primary and secondary endpoints. The latest results from the identical cohort of patients were considered. If disagreements arose, a third researcher (Jiuwei Cui) made the final decisions.

### Quality Assessment

The risk of bias of an individual study was assessed using the Cochrane Risk of Bias Tool, built into the Review Manager software (version 5.3), using the following items: random sequence generation, allocation concealment, blinding of participants and personnel, blinding of outcome assessment, incomplete outcome data, selective outcome reporting, and other bias sources. Each item was marked as low, high, or unclear risk of bias.

### Statistical Analyses

We performed this meta-analysis using Stata/SE software (College station, TX, USA) (version 15.0). The pooled hazard ratio (HR) and 95% CI were generated to analyze the PFS advantage, while the pooled OR and 95% CI were calculated to compare ORR, DCR, and the rate of grades 3–5 AEs. *I*^2^ and χ^2^ tests were utilized to assess the heterogeneity among the included studies. Either *I*^2^ > 50% or *P* < 0.1 illustrated significant heterogeneity. Accordingly, the randomized-effects model was employed; otherwise, the fixed-effects model was employed. Statistical differences were defined as *P* < 0.05. Sensitivity analyses of PFS, ORR, DCR, OS, and grades 3–5 AEs were conducted to detect the robustness of the meta-analysis results. Because the number of articles is <10, we did not conduct publication bias tests.

## Results

### Search Results

**Figure 1:**
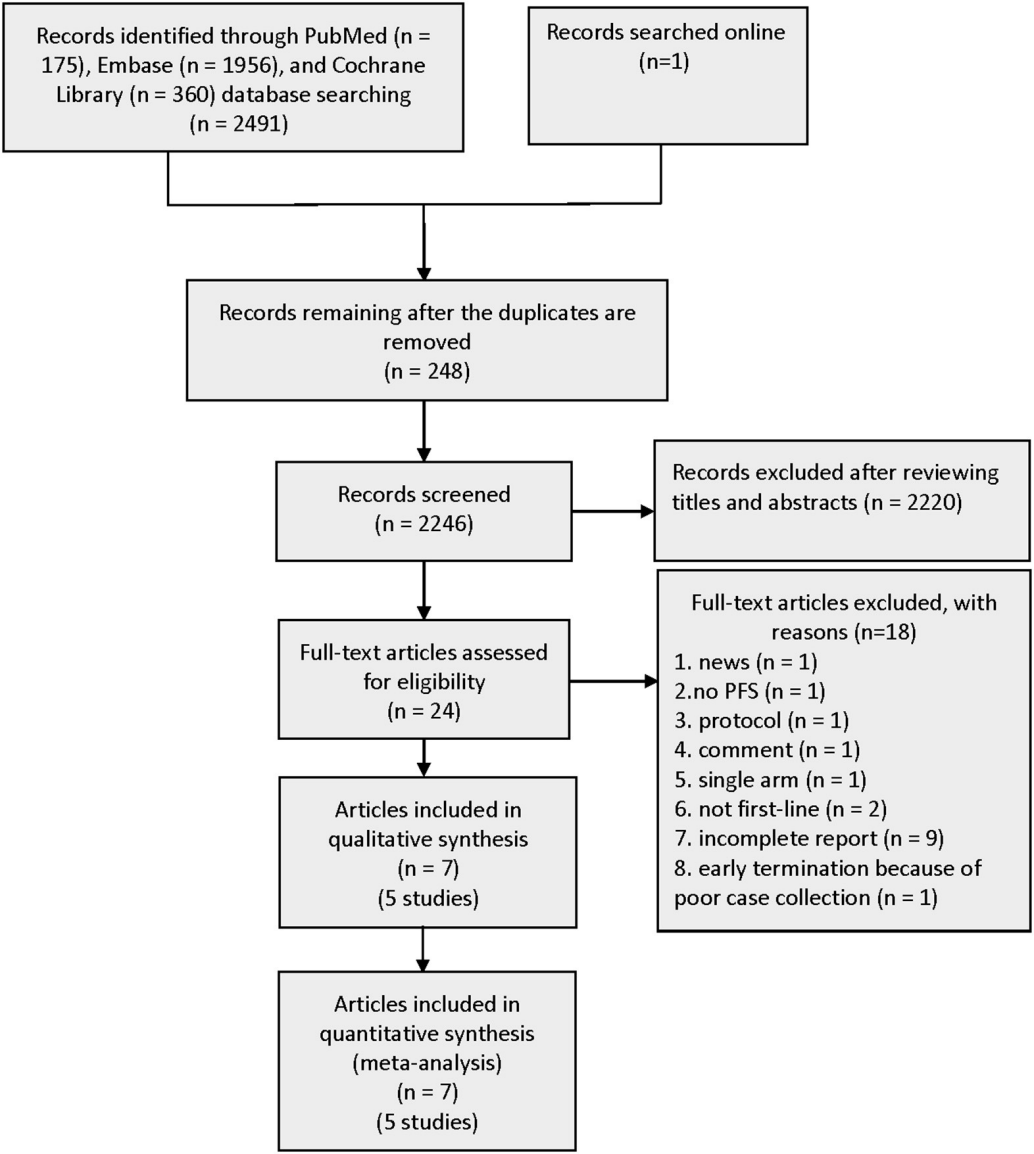
Flowchart of the study selection process.

A total of 2,491 records were identified from three pivotal databases—PubMed, EMBASE, and Cochrane Library. The data from the CTONG 1,509 study presented at the 2019 European Society for Medical Oncology (ESMO) Congress were also made available online. A total of 248 duplicate records were removed from the 2,492 records. Subsequently, by screening titles and abstracts, 24 promising publications were fully reviewed. Aligning with the predefined inclusion criteria, five RCTs involving 1,226 patients were used for the analyses (Figure 1). The excluded publications include many trial protocols (interventions include the first-generation EGFR-TKI gefitinib in combination with bevacizumab or anlotinib or fruquintinib, second-generation EGFR-TKI afatinib in combination with bevacizumab, and third-generation EGFR-TKI osimertinib combined with bevacizumab).

### Main Characteristics and Quality Evaluation

**Figure 2:**
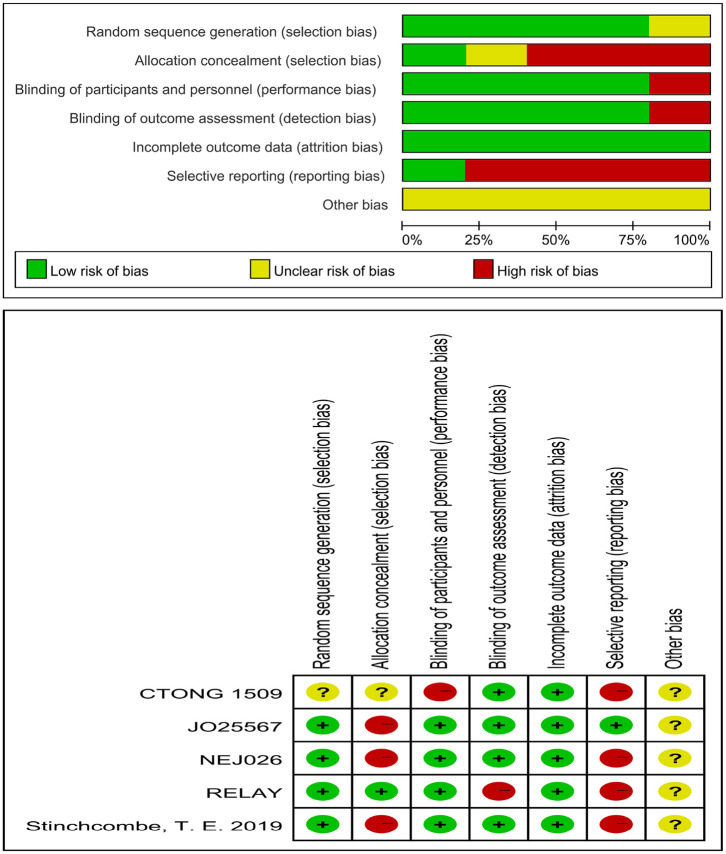
Risk of bias of the included studies.

Five journal articles (27–31), one conference abstract (32), and an oral presentation at the 2019 ESMO congress (33) met the inclusion criteria, including two Japanese studies, one Chinese study, one American study, and one international multicenter study. Three trials included brain metastatic patients. The *EGFR* mutation types for patients were *exon 19* deletions and *21 Leu858Arg* mutations. The intervention for the experimental group was the first-generation EGFR-TKI erlotinib plus bevacizumab (anti-VEGF antibody) (27, 30, 31, 33) or erlotinib plus ramucirumab (anti-VEGFR antibody) (29), whereas the control group was administered erlotinib alone or erlotinib with placebo. Table 1 lists the primary features of all included studies. The Cochrane Risk of Bias Tool was employed to appraise the research quality (Figure 2).

**Table 1:** Main characteristics of the included studies.

**Study (phase, area)**	**Sample size (no.)**	**Median age (years)**	**Male/female (%)**	**Disease stage**	**Treatment**	**PFS (months)**	**ORR (%)**	**DCR (%)**	**Grade ≥3 AEs (%)**	**OS (months)**
JO25567 (II, Japan)	75	67	40/60	IIIB/IV or recurrent	Erlotinib 150 mg/d + bevacizumab 15 mg/kg Q3w	16.0	69	99	91	47.0
	77	67	34/66		Erlotinib 150 mg/d	9.7	64	88	53	47.4
RELAY (III, worldwide)	224	65	37/63	IV or recurrent	Erlotinib 150 mg/d + ramucirumab 10 mg/kg Q2w	19.4	76	95	72	NR
	225	64	37/63		Erlotinib 150 mg/d + placebo Q2w	12.4	75	96	54	
NEJ026 (III, Japan)	112	67	37/63	IIIB/IV or recurrent	Erlotinib 150 mg/d + bevacizumab 15 mg/kg Q3w	16.9	72	95	88	NR
	114	68	35/65		Erlotinib 150 mg/d	13.3	66	96	46	
Stinchcombe, 2019 (II, US)	43	65	28/72	IV	Erlotinib 150 mg/d + bevacizumab 15 mg/kg Q3w	17.9	81	NR	NR	32.4
	45	63	31/69							
					Erlotinib 150 mg/d	13.5	83			50.6
CTONG 1509 (III, China)	157	57	38.2/61.8	IIIB/IV or recurrent	Erlotinib 150 mg/d + bevacizumab 15 mg/kg Q3w	18.0	86.3	95.9	53.5	NR
	154	59	37.7/62.3							
					Erlotinib 150 mg/d	11.3	84.7	96.5	25.5	

*PFS, progression-free survival; OS, overall survival; ORR, overall response rate; DCR, disease control rate; DOR, duration of response; AEs, adverse events; NR, not reported*.

### Efficacy

**Figure 3:**
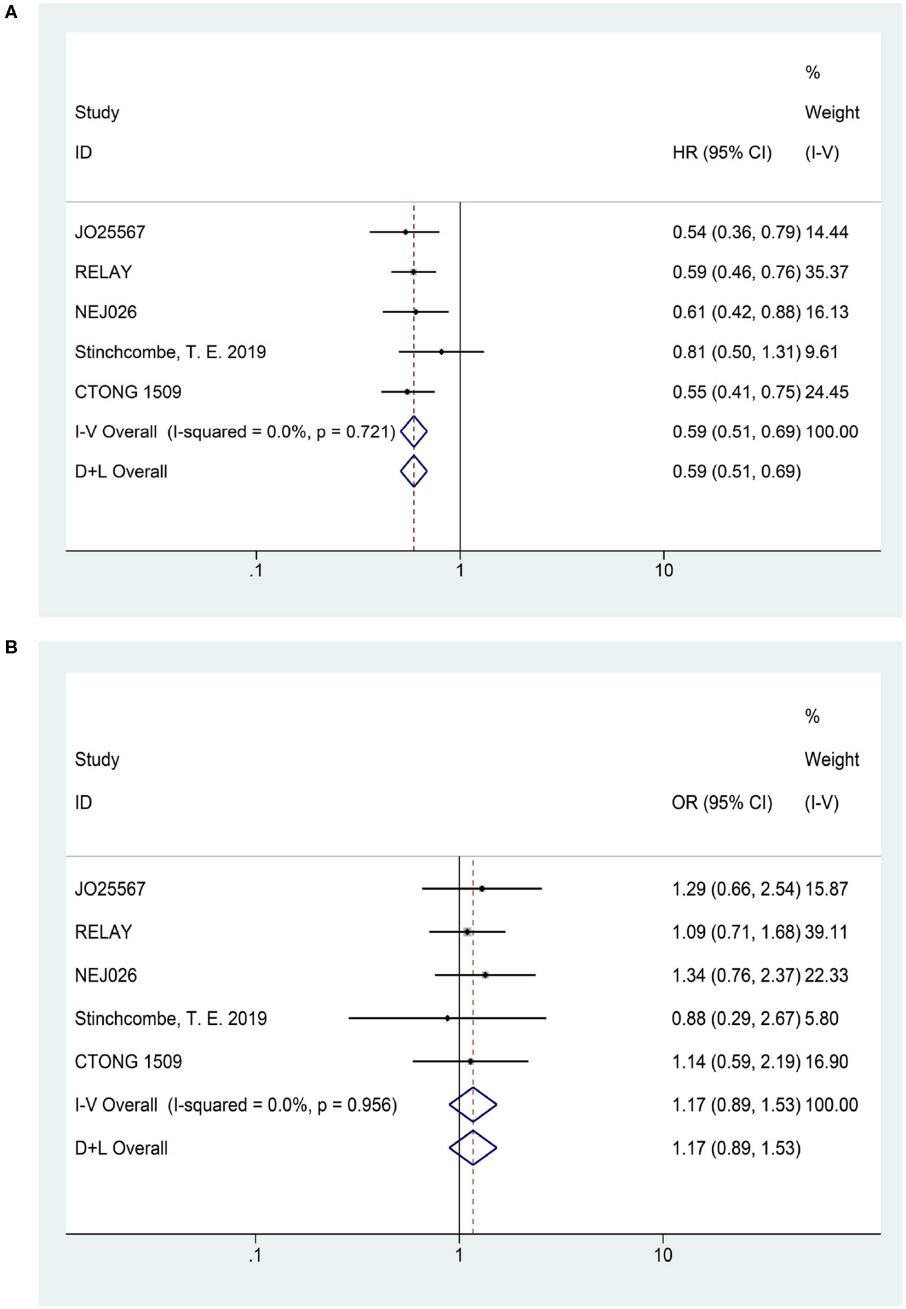
Forest plots of the PFS **(A)** and ORR **(B)** that are associated with erlotinib plus antiangiogenic agents vs. erlotinib.

In terms of PFS (heterogeneity: *P* = 0.721, *I*^2^ = 0), the pooled results from five studies demonstrated that erlotinib plus anti-VEGF/anti-VEGFR antibody could improve PFS compared to the erlotinib monotherapy (HR = 0.59, 95% CI = 0.51–0.69, *P* = 0.000; Figure 3A).

Five articles reported the ORR (heterogeneity: *P* = 0.956, *I*^2^ = 0). The pooled results suggested that there was no significant difference in ORR between combination therapy and erlotinib alone (OR = 1.17, 95% CI = 0.89–1.53, *P* = 0.258; Figure 3B).

**Figure 4:**
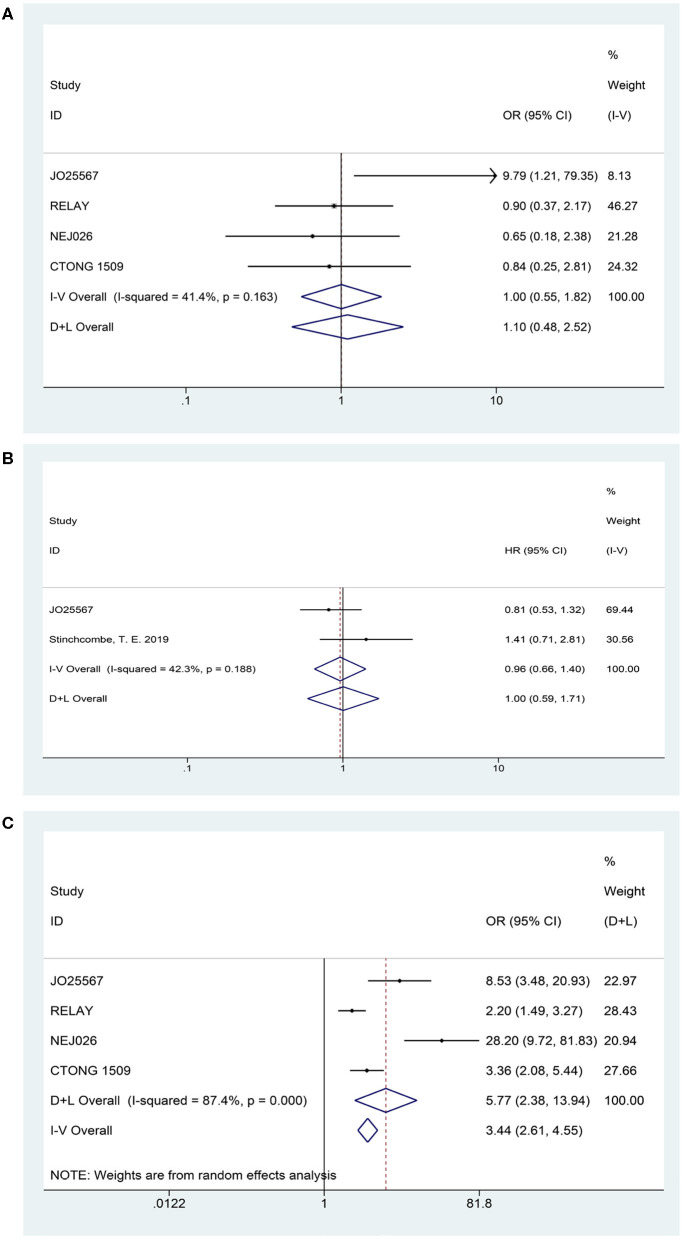
Forest plots of the DCR **(A)**, OS **(B)**, and grade ≥3 AEs **(C)** that are associated with erlotinib plus antiangiogenic agents vs. erlotinib.

Four articles presented DCR (heterogeneity: *P* = 0.163, *I*^2^ = 41.4%). Patients could not receive more benefit from erlotinib plus anti-VEGF therapy than erlotinib monotherapy (OR = 1.00, 95% CI = 0.55–1.82, *P* = 0.989; Figure 4A).

Two studies published their OS findings (heterogeneity: *P* = 0.178, *I*^2^ = 44.9%). However, current data have not demonstrated that patients experienced an OS prolongation benefit when treated with combination therapy (HR = 0.94, 95% CI = 0.66–1.35, *P* = 0.745; Figure 4B).

### Safety

**Figure 5:**
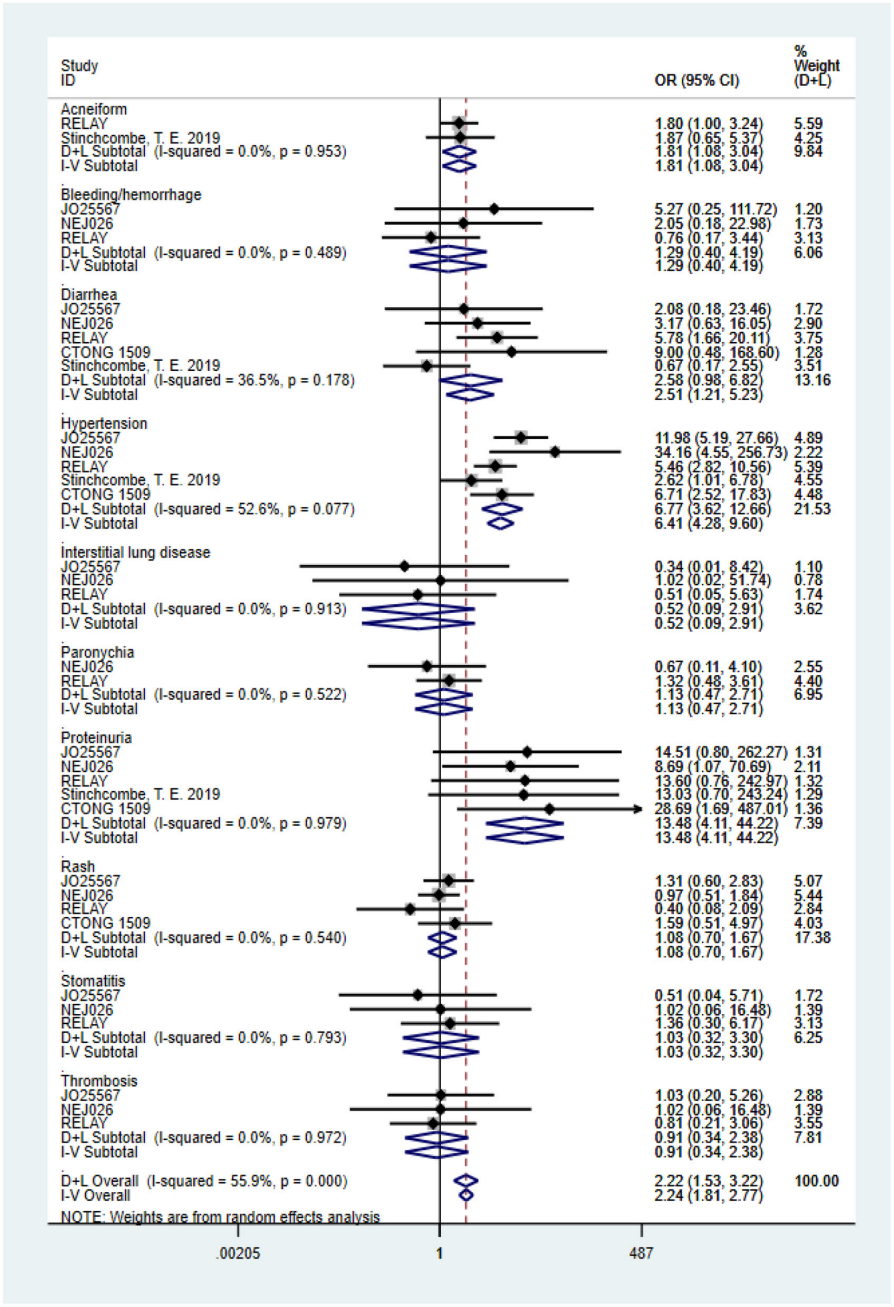
Forest plots of the 10 primarily mentioned grade ≥3 AEs that are associated with erlotinib plus antiangiogenic agents vs. erlotinib.

Based on the total number of patients who experienced AEs, we analyzed the difference in the incidence rate of grades 3–5 AEs between combination therapy and erlotinib alone. The data for the overall grades 3–5 AEs originated from four trials (heterogeneity: *P* = 0.000, *I*^2^ = 87.4%). Results demonstrated that the dual-blocking strategy caused substantial increase in grades 3–5 AEs relative to erlotinib monotherapy (OR = 5.772, 95% CI = 2.38–13.94, and *P* = 0.000; Figure 4C). Additionally, we performed subgroup analysis of the 10 primarily mentioned AEs and found that combination therapy led to higher incidence of diarrhea (OR = 2.51, 95% CI = 1.21–5.23, *P* = 0.014), acneiform (OR = 1.82, 95% CI = 1.08–3.04, *P* = 0.023), hypertension (OR = 6.77, 95% CI = 3.62–12.66, *P* = 0.000), and proteinuria (OR = 13.48, 95% CI = 4.11–44.22, *P* = 0.000; Figure 5).

### Subgroup Analysis

**Figure 6:**
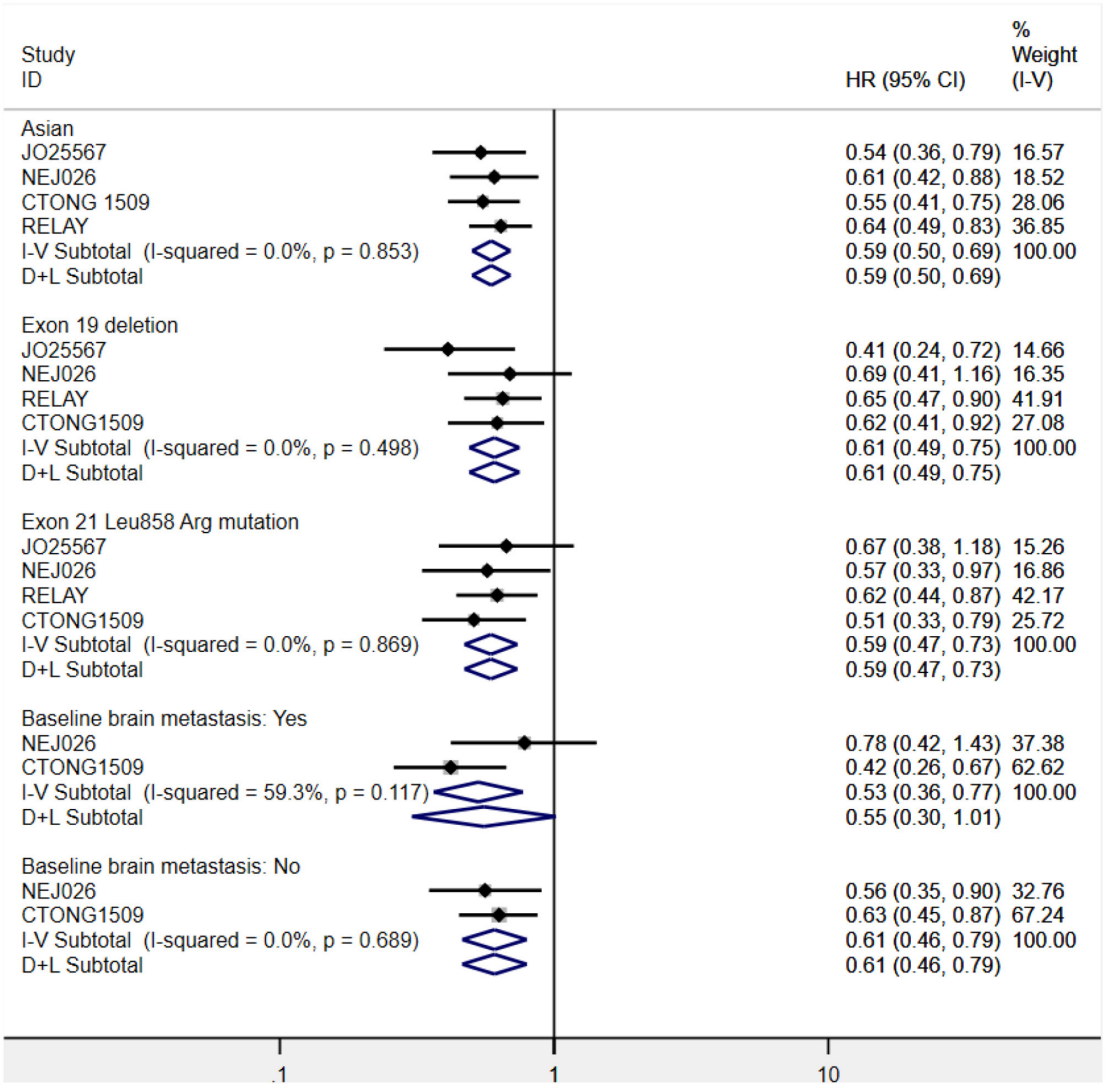
Forest plots of the PFS subgroup of Asian patients, *exon 19* deletions and *21 Leu858Arg* mutations, and brain metastasis status at baseline that are associated with erlotinib plus antiangiogenic agents vs. erlotinib.

Considering the influence of race on efficacy, we first conducted a subgroup analysis of the patient population. The ethnic stratification of each study included Japanese (JO25567 and NEJ026 study), Chinese (CTONG 1509 study), East Asian, and other races (RELAY study), and white and nonwhite (phase II study of the United States). Accordingly, we grouped the total population into two categories: Asian and non-Asian. A phase II study in the United States only reported the intergroup HR of the white and nonwhite groups (HR = 0.86, 95% CI = 0.41–1.81), whereas the RELAY study reported the HR of East Asian patients and other patients (0.64 and 0.61, respectively). Therefore, only the pooled PFS value of Asian patients was retrieved. The result showed that the risk of disease progression of Asian patients in the combined treatment group was reduced by 41% (HR = 0.59, 95% CI = 0.50–0.69, *P* = 0.000), which is consistent with the result for the overall populations. To ascertain the possible *EGFR* mutation type–mediated discrepancies in treatment efficacy, we performed the subgroup analysis of *exon 19* deletions and *21 Leu858Arg* mutations. Combination therapy yielded better PFS benefits irrespective of the mutation type. Further, disease progression risk was reduced by 39% in the *exon 19* deletions subgroup (HR = 0.61, 95% CI = 0.49–0.75, *P* = 0.000) and 41% in the *21 Leu858Arg* mutation subgroup (HR = 0.59, 95% CI = 0.47–0.73, *P* = 0.000). Furthermore, we analyzed the pooled PFS for the brain metastasis subgroups. One study reported only the HR between groups with and without brain metastases. The pooled result of the two trials showed that erlotinib plus bevacizumab had better efficacy for patients without brain metastasis at baseline (HR = 0.61, 95% CI = 0.46–0.79, *P*= 0.000). For patients with brain metastases at baseline, the current data did not indicate there was a difference between the two regimens (HR = 0.55, 95% CI = 0.30–1.01, *P*= 0.001; Figure 6).

### Sensitivity Analyses

The pooled PFS, ORR, DCR, AE, and PFS subgroup analyses of the Asian population and two mutation types were all robust, with concordant conclusions derived from the sensitivity analysis and no estimates beyond the 95% CIs. However, the PFS subgroup of patients with brain metastasis and OS results were not persuasive. This might be due to the small number of eligible trials for analyses. In terms of overall grades 3–5 AEs, the RELAY study dramatically differed from other studies. Additionally, replacing the effect model did not render any opposite findings except for the incidence of diarrhea and the PFS subgroup of brain metastatic patients (Figures S1–S6; Figures 3–6).

## Discussion

To the best of our knowledge, this is the first meta-analysis to compare the efficacy and safety of EGFR-TKIs plus antiangiogenesis therapy to that of EGFR-TKIs alone as a first-line treatment for NSCLC. Through systematic and comprehensive literature research, we found multiple trials that have sought to determine the efficacy of EGFR-TKIs combined with antiangiogenic drugs as a first-line treatment (including the first-, second-, and third-generation EGFR-TKIs combined with anti-VEGF/VEGFR monoclonal antibodies or small molecule TKIs) (34–38). However, only RCTs on the first-generation EGFR-TKI erlotinib combined with anti-VEGF/VEGFR antibodies reported the endpoint results. Analyses of these large-scale, high-quality phase II/III RCTs illustrated that erlotinib combined with bevacizumab or ramucirumab had the advantage of PFS extension relative to erlotinib. For Asian patients, combination therapy had evident PFS advantages. Patients with *exon 19* deletions and *21 Leu858Arg* mutations could gain similar PFS benefits from dual-blocking therapy. However, grade 3–5 AEs, especially acneiform, hypertension, proteinuria, and diarrhea, were increased in the combination group.

We proved that the first-generation EGFR-TKI erlotinib plus anti-VEGF/VEGFR antibody has PFS advantage over erlotinib alone for *EGFR*-mutant advanced NSCLC patients. This meta-analysis provides evidence to support the use of EGFR-TKIs combined with antiangiogenesis therapy as a first-line treatment for *EGFR*-mutant NSCLC patients. Among the five trials included in our meta-analysis, PFS prolongation ranged from 3.6 to 7.0 months in the combination arm (27, 29–31, 33). A single-arm phase II study in Japan suggested that the first-generation EGFR-TKI gefitinib combined with bevacizumab as a first-line therapy for *EGFR*-mutant NSCLC patients increased PFS by 4–5 months relative to the historic EGFR-TKI monotherapy (39). In the *EGFR*-mutant subgroup of the phase III BeTa trial, erlotinib plus bevacizumab increased the PFS by 7.2 months compared to erlotinib for advanced NSCLC, which did not respond to standard first-line chemotherapy (40). A retrospective study revealed that erlotinib/gefitinib plus bevacizumab yielded PFS of 16.0 months in the first-line setting, which is similar to that in the JO25567 study (41). Notably, the FLAURA study confirmed the superiority of the third-generation EGFR-TKI osimertinib over other EGFR-TKIs as a first-line treatment for *EGFR*-sensitive mutations in NSCLC patients, with PFS of 18.9 months and OS of 38.6 months (7, 42). As a result, the following questions arose: what are the differences between osimertinib and first-generation EGFR-TKIs plus antivascular drugs, in terms of efficacy, and could osimertinib plus antivascular drugs improve survival, compared to osimertinib alone? The ongoing single-arm phase I/II clinical trial of osimertinib plus bevacizumab (34) and the phase II RCT of osimertinib plus bevacizumab vs. osimertinib (43) are expected to answer these questions. Osimertinib is effective for patients with *T790M* mutation in *EGFR exon 20*, which accounts for 40–60% of resistant mutations (44–46). Epidermal growth factor receptor–TKIs plus antiangiogenic agents can delay the occurrence of resistance (15). If the combination of first-generation EGFR-TKIs and antiangiogenic agents could yield a similar survival benefit relative to osimertinib in advanced patients with *EGFR*-sensitive mutations, first-generation TKIs plus antiangiogenic therapy could be employed in the first-line setting, and osimertinib could be considered a subsequent option when *T790M* mutation occurred, which is expected to maximize patient survival. Moreover, a variety of EGFR-TKIs and different classes of antivascular drugs currently exist. Are there any differences in the effects of different combinations of these drugs? More research data, head-to-head comparisons, and network meta-analyses are needed to answer this question. In addition, in the case of multiple treatment options, a cost-effectiveness analysis is of marked importance for therapy selection.

Notably, the efficacy of combination therapy may be discrepant in different populations. Patients recruited in the JO25567 and NEJ026 studies were all Japanese. Further, the PFS was found to significantly increase in the combination group (27, 30). The CTONG 1509 study focused on Chinese patients and achieved positive results (33). In the RELAY study, most of the population was East Asians (75%), and their PFS was remarkably increased (HR = 0.64, 95% CI = 0.49–0.83). However, no statistically significant difference in PFS was found in other populations (white, American Indian or Alaska Native, black or African–American, or missing) (HR = 0.61, 95% CI = 0.36–1.01) (29). The phase II study in the United States revealed no difference in PFS between white and nonwhite subgroups (African American, Asian, not available) (HR = 0.86, 95% CI = 0.41–1.81) in two groups and increased risk of death in the combination group (31). As the existing data format for non-Asian patients is limited, we analyzed data from Asian patients and found that combination therapy significantly improved the PFS of these patients to the same extent as that in general populations, with equal HR. Therefore, Asian patients can benefit from combination therapy; however, the data for other races remain insufficient to draw relevant conclusions. It is hoped that future studies with large sample sizes will elucidate the effect of race on efficacy.

*Exon 19* deletions and *21 Leu858Arg* mutations account for 50 and 40% of all *EGFR* gene mutation types, respectively (47). Patients that harbor these two most common types of driving mutations are sensitive to EGFR-TKIs (3). A meta-analysis revealed that patients with *exon 19* deletions had a better prognosis than those with *21 Leu858Arg* mutations (48). However, our generated data revealed similar PFS benefits between the two mutation types in the combination arm. The risk of disease progression was reduced by 39 and 41% for *exon 19* deletions and *21 Leu858Arg*, respectively. Three phase III trials (NET026, RELAY, and CTONG 1509) demonstrated that patients with *exon 21 Leu858Arg* mutations had greater reduction in disease progression risk than those with *exon 19* deletions in the combination group (29, 30, 33), whereas only one phase II trial (JO25567) showed contrasting results (27). In the subgroup analysis in the CTONG 1509 study, the PFS of patients with *exon 21 Leu858Arg* mutations was 19.5 months in the erlotinib plus bevacizumab group (33), which is the longest PFS observed to date. This result exceeded the 14.4 months PFS of patients receiving monotherapy with the third-generation TKI osimertinib (7). Apparently, the combination of erlotinib plus anti-VEGF/VEGFR agents is more beneficial for patients with *21 Leu858Arg* mutations. Therefore, the synergistic antiproliferative effects of EGFR-TKIs and antiangiogenic treatment might eliminate the prognosis differences caused by genetic mutations. Nevertheless, we await the OS data to verify this hypothesis.

Patients with brain metastasis have poor prognosis. The probability of brain metastasis in patients harboring *EGFR* mutations is three-fold greater than that in patients without *EGFR* mutations. Among the five studies included in this meta-analysis, only three (NEJ026, CTONG 1509, and Stinchcombe, T. E. 2019) (30, 31, 33) recruited patients with brain metastasis at baseline. The phase II trial in the United States (31) reported that baseline brain metastasis status was not related to PFS (HR = 1.72, 95% CI = 0.92–3.23). Although pooled PFS of patients with brain metastasis at baseline was not statistically different, a tendency of PFS benefit could be observed using combination therapy. In the CTONG 1509 study, combination therapy reduced the risk of progression by half in brain metastatic patients (33). Another study demonstrated that the intracranial tumor control rate in brain metastatic patients in the erlotinib and bevacizumab combination group was almost twice that in the erlotinib monotherapy group (49). However, current data are limited to reach a definitive conclusion. Future studies of EGFR-TKIs combined with antiangiogenic agents could consider including patients with brain metastasis.

Objective response rate and DCR are both implicated in tumor response to drugs. In the combination group, the proportion of patients who reached complete response was low, with only 3 of the 224 patients (1%) in the RELAY study reaching complete response (29). According to our meta-analysis results, there were no differences in ORR and DCR improvement between erlotinib plus anti-VEGF/VEGFR drugs and erlotinib alone. Overall survival extension is the ultimate goal of antitumor therapy. Our meta-analysis result of OS was negative, which might be attributed to the limited data from the two phase II RCTs. After OS is reached in the other three phase III RCTs, we will update the pooled results. Overall survival is affected by different factors, such as the statistical design of the trials, posterior treatments, and complicated biological mechanisms. A preclinical study found that anti-VEGF therapy increased tumor hypoxia, hypoxia-inducible factor 1α, and c-Met activation and facilitated cancer invasion and metastasis (50). More in-depth research and clinical data are required to better assess the long-term benefits of combination therapy.

Furthermore, safety should be considered for treatment evaluation. Adverse events, especially grades 3–5 AEs, remarkably impair patients' treatment compliance and quality of life, thereby indirectly undermining antitumor efficacy. Our pooled results demonstrate that acneiform, hypertension, proteinuria, and diarrhea evidently increased in the combination group. However, the difference in side effects caused by different combinations of EGFR-TKI and antivascular drugs is unclear. Following the publication of more trial results, a network meta-analysis may be a good approach to comprehensively compare AEs. Moreover, we found that increased AEs were associated with better PFS. Concurrently, two other studies found that antiangiogenesis-related AEs were associated with prolonged PFS and OS (51, 52). Therefore, on the basis of close monitoring, timely identification, and instant management of AEs, EGFR-TKI combined with anti-VEGF/VEGFR agents may be a favorable strategy in the first-line setting for *EGFR* mutant NSCLC.

This study had several limitations. First, the reliability of our results is reduced by the limited number of studies. Second, some trials are ongoing and the final OS data have not been announced. Third, insufficient data on brain metastasis and race led to unreliable analyses results. Fourth, a test of publication bias was not performed as the number of included trials was fewer than 10. Several clinical investigations that aim to explore the role of EGFR-TKIs plus antiangiogenic drugs in the first-line setting for advanced NSCLC are underway (35–38, 43). Thus, we anticipate the publication of new data and will update the meta-analysis results to better guide clinical decisions.

## Conclusions

In summary, this meta-analysis demonstrates that the first-generation EGFR-TKI erlotinib plus antiangiogenesis therapy yields more benefits in terms of PFS when administered as a first-line therapy for *EGFR*-sensitive mutations NSCLC than erlotinib alone. The combination therapy resulted in evident PFS benefits for Asian patients. Further, *exon 19* deletions and *21 Leu858Arg* mutations were found to gain similar benefits from the administration of combination therapy. Patients with brain metastases at baseline also tended to display PFS benefits. The combination strategy causes a higher incidence of grades 3–5 diarrhea, acneiform, hypertension, and proteinuria. Our work provided evidence to support the addition of antiangiogenesis agents to anti-EGFR therapy for use as a first-line treatment for *EGFR*-mutant NSCLC patients. Several studies on combination therapy are ongoing, and an update of the pooled results is required after the data are updated to better determine the practical application of EGFR-TKIs in combination with antiangiogenic agents.

## Data Availability Statement

All datasets analyzed for this study are included in the article/Supplementary Material.

## Author Contributions

FC and JC conceived and designed the study. FC and NC performed the literature search, data extraction, quality assessment of the included studies, and statistical analysis. FC wrote the paper. NC, YY, and JC reviewed and edited the manuscript. All authors read and approved the manuscript.

## Conflict of Interest

The authors declare that the research was conducted in the absence of any commercial or financial relationships that could be construed as a potential conflict of interest.

## Supplementary Material

Table S1 PRISMA 2009 checklist.

Figure S1 Sensitivity analysis of PFS **(A)** and ORR **(B)** associated with erlotinib plus antiangiogenic agents vs. erlotinib.

Figure S2 Sensitivity analysis of DCR **(A)**, OS **(B)**, and the grade ≥3 AEs **(C)** associated with erlotinib plus antiangiogenic agent vs. erlotinib.

Figure S3 Sensitivity analysis of diarrhea **(A)**, acneiform **(B)**, hypertension **(C)**, and proteinuria **(D)** associated with erlotinib plus antiangiogenic agent vs. erlotinib.

Figure S4 Sensitivity analysis of the Asian population and its association with erlotinib plus antiangiogenic agent vs. erlotinib.

Figure S5 Sensitivity analysis of *Exon 19* deletions **(A)** and *Exon 21 Leu858Arg* mutations **(B)** and their association with erlotinib plus antiangiogenic agents vs. erlotinib.

Figure S6 Sensitivity analysis of brain metastasis at baseline **(A)** and no brain metastases at baseline **(B)** and their association with erlotinib plus antiangiogenic agent vs. erlotinib.
